# Validation of a New and Straightforward Algorithm to Evaluate Signal Quality during ECG Monitoring with Wearable Devices Used in a Clinical Setting

**DOI:** 10.3390/bioengineering11030222

**Published:** 2024-02-26

**Authors:** Luca Neri, Ilaria Gallelli, Massimo Dall’Olio, Jessica Lago, Claudio Borghi, Igor Diemberger, Ivan Corazza

**Affiliations:** 1 Division of Cardiology, Department of Medicine, Johns Hopkins University, Baltimore, MD 21218, USA; 2 Department of Medical and Surgical Sciences, University of Bologna, 40126 Bologna, Italy; 3 IRCCS AOU, Policlinico di S. Orsola, 40138 Bologna, Italy

**Keywords:** wearable devices, clinical reliability, ECG, low-cost technology, signal quality evaluation

## Abstract

Background: Wearable devices represent a new approach for monitoring key clinical parameters, such as ECG signals, for research and health purposes. These devices could outcompete medical devices in terms of affordability and use in out-clinic settings, allowing remote monitoring. The major limitation, especially when compared to implantable devices, is the presence of artifacts. Several authors reported a relevant percentage of recording time with poor/unusable traces for ECG, potentially hampering the use of these devices for this purpose. For this reason, it is of the utmost importance to develop a simple and inexpensive system enabling the user of the wearable devices to have immediate feedback on the quality of the acquired signal, allowing for real-time correction. Methods: A simple algorithm that can work in real time to verify the quality of the ECG signal (acceptable and unacceptable) was validated. Based on simple statistical parameters, the algorithm was blindly tested by comparison with ECG tracings previously classified by two expert cardiologists. Results: The classifications of 7200 10s-signal samples acquired on 20 patients with a commercial wearable ECG monitor were compared. The algorithm has an overall efficiency of approximately 95%, with a sensitivity of 94.7% and a specificity of 95.3%. Conclusions: The results demonstrate that even a simple algorithm can be used to classify signal coarseness, and this could allow real-time intervention by the subject or the technician.

## 1. Introduction

Over the past decade, the healthcare field has undergone rapid transformation, primarily driven by the development and introduction of new technologies embedded in wearable devices [1,2,3]. These devices can collect real-time data, such as the electrocardiogram (ECG), enabling prolonged non-invasive monitoring, potentially helpful in monitoring clinical parameters and the early detection of acute events [4,5]. However, a significant challenge lies in ensuring optimal signal quality [6,7], often compromised by several factors, including movement, sweating, and displacement of sensors, caused by suboptimal electrode adherence and stability [8].

These limitations become particularly relevant when wearable sensors, chosen for their simplicity, cost-effectiveness, and prolonged monitoring capabilities, are considered as alternatives to traditional, more reliable, and more expensive medical equipment [9]. Several authors have proposed this for in-hospital monitoring (to increase the number of monitored patients at a reduced cost [10]) and for out-of-hospital scenarios, where wearable instrumentation could significantly decrease the healthcare burden [11,12,13]. Cost reduction is not the only potential outcome of these technologies, since they can improve access to a broader group of patients, mitigating disparities [14]. The benefits in terms of prevention and early diagnosis are potentially relevant. The COVID-19 pandemic served as a powerful accelerator of this process, with increased interest from healthcare professionals and a rise in the number of devices in the market. In this specific scenario, remote monitoring played an additional role in controlling the spreading of the infection by minimizing the need for patients to access hospitals physically, consequently reducing healthcare expenses [15,16,17].

Today, dedicated signal acquisition software automatically classifies signal quality [18] with traditional and novel algorithms (for example, by implanting artificial intelligence—AI) [19,20,21,22,23,24]. These methods have variable sensitivity and specificity, with an overall accuracy usually >90% [6]. However, these methods are applied during post-processing when the ECG signal has already been acquired. Real-time verification of signal quality becomes crucial when we want to rely only on these technologies [23,25]. Real-time verification would improve users’ compliance, allowing them to check for noisy signals, enabling sensor repositioning and ensuring better signal quality. This approach could improve electrocardiographic diagnosis, enhancing device efficiency and avoiding the potential need for repeating the evaluation. It is evident that achieving this result requires the implementation of simple algorithms that operate in real time and provide immediate feedback to the device user.

In this study, we developed a simple, inexpensive, and lightweight algorithm capable of distinguishing between a good quality and unreadable ECG in real time. ECG was acquired by a commercial device and preventively classified by two expert cardiologists. Then, ECG signals were automatically classified by the proposed algorithm. Statistical comparison in terms of sensitivity and specificity was carried out [26].

## 2. Materials and Methods

### 2.1. Background

The current approach to improving signal quality involves heavy filtering, albeit with the unintended consequence of diminishing or distorting valuable information and altering the signal morphology. Addressing this challenge is particularly intricate due to the inherent nature of these devices, designed for continuous and long-term monitoring during users’ daily activities. This work proposes a novel strategy, as detailed herein, which involves developing streamlined algorithms for real-time ECG analysis. These algorithms are designed to be easily integrated, ensuring swift calculations for generating ECG classifications. This approach aims to accomplish the following:

1. Enhance post-processing by extracting relevant signal segments selectively, improving signal processing efficiency;
2. Implement real-time algorithms that provide acoustic or haptic feedback when noise compromises the signal. This possibility will empower users to take prompt corrective actions, such as adjusting electrode placement, thus potentially preventing the recording of hours of noisy signals.

The primary focus of this study is the offline validation of an algorithm designed for signal classification into the following two categories:

1. Clean/good quality/acceptable: All primary waves constituting the ECG (P, QRS, and T) are visible.
2. Compromised/bad/unacceptable: The signal is partially unreadable, making it impossible to correctly identify the QRS complex, and P and Q waves.

The main idea of the proposed solution is based on a simple assumption: for a wearable device, the noise sources are mainly represented by muscular artifacts and by displacement or loss of adhesion of the electrodes. In both cases, the noise occurs with waves of variable amplitude (often near saturation) and variable frequency. Therefore, we can state that a readable ECG is characterized by the following:

1. Limited variability in the isoelectric line;
2. A limited range of signal variation (maximum (max)–minimum (min) values);
3. A limited statistical variability (quantified through the standard deviation, SD).

**Figure 1:**
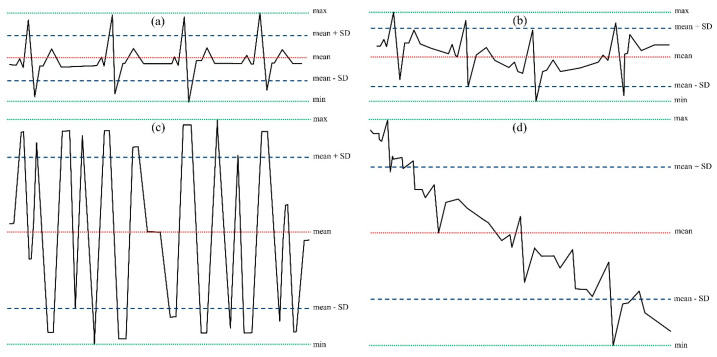
Example of electrocardiographic signals: (**a**) clean signal; (**b**) signal with a slight fluctuation of the isoelectric line; (**c**) noise due to electrodes movements and loss of adhesion; (**d**) signal with a high isoelectric line variability and muscle artifacts. The dotted green lines represent the maximum (max) and minimum (min) values, the red line is the mean value (mean), and the dashed blue lines represent the mean values ± standard deviations (SD).

Figure 1 shows an example of the typical morphologies of the ECG signal.

A noisy signal presents a wide range of short-time variability (max-min) with values sometimes close to saturation and a higher standard deviation. By choosing appropriate thresholds, it is possible to discriminate the trace based on simple parameters easily calculable in real-time: mean value (mean), standard deviation (S.D.), maximum value (max), and minimum value (min). The choice of thresholds can be made in three ways: (a) manually, allowing the operator to extract these values from a template selected directly on the ECG; (b) automatically, using a dedicated algorithm capable of extracting the template from the signal (therefore performed with the patient in stationary conditions); (c) with a pre-set template, based on the technical characteristics of the device (amplification, A.D. conversion range, electrodes positions). For the validation of the algorithm, the second proposed solution was chosen.

### 2.2. Experimental Design

For the validation of the algorithm, ECG tracings of 20 patients (age: 55.3 years (SD 21.5), sex: 10 females and 10 males) were provided with a t-shirt equipped with polymer-based sensors (YouCare, AccYouRate Group S.p.A., L’Aquila, Italy) and capable of recording a differential ECG lead between two electrodes located close to the diaphragm, just below the pectoralis major muscle. A third electrode is positioned on the back of the chest belt, and it has the function of the right leg lead used to reduce the noise and artifacts present on the other two electrodes. The embedded ADC has an 18bit resolution and an input range of ±500 mV. ECG data are sent to the patient’s smartphone (with dedicated software) and recorded.

The patients were enrolled within the protocol number 156/2022/Disp/AOUBo (approved by the Ethics Committee Area Vasta Emilia Centro—CE-AVEC—Bologna). The study was conducted following the Declaration of Helsinki, and each participant provided written informed consent after being informed about the nature and potential risks of the studies. Since this work aimed to validate a signal quality classification algorithm, neither inclusion nor exclusion criteria were considered.

The t-shirt was used as a Holter recorder, mounted, and activated in a clinical setting, and patients were free to move and conduct the actions of their everyday life. The first hour of monitoring was extracted from each patient. As such, for algorithm validation, 20 h of ECG signals were considered.

Signal quality assessment was made by two expert cardiologists who marked the acceptable parts of the signal (clearly identifiable P, QRS, and T waves) and those considered compromised due to the difficulty of identifying even the QRS wave of multiple adjacent complexes. Moreover, a signal with no P wave but with definite R.R. intervals and a Q wave was considered acceptable in the case of atrial fibrillation.

The manual classification was performed using Anscovery software (SparkBio Srl, San Lazzaro di Savena, Bologna, Italy).

Cardiologists worked on the whole traces and performed the classifications, putting two digital markers at the beginning and end of each classified period. The length of each period can be different and as long as at least one beat (resolution). To make their classification comparable (with the one performed by the algorithm), consecutive samples of 10 s, starting at the beginning of the recording, were considered. Each 10 s sample was labeled “acceptable” or “unacceptable” depending on the prevalent classification (lasting at least 5 s) over each sample. At the end of the process, 360 samples (10 s windows) were extracted together with their category (“acceptable” or “unacceptable”) for each patient.

Manual classification was then erased, and ECG signals were processed with the automatic algorithm.

The algorithm is composed of the following four main blocks:

(1) Reference values calculation: search for the first stable signal period (constant isoelectric line and amplitude variability less than 70% of the maximal acquisition range for at least 10 s). Maximum (max_t_), minimum (min_t_), and standard deviation (SD_t_) values were used as a reference for the subsequent steps.
(2) Starting from the trace starting point, selection of consecutive signal 2 s windows for quality evaluation. Calculation of maximum (max_w_), minimum (min_w_), and standard deviation (SD_w_) over the 2 s window;
(3) Comparison between the maximum (max_w_) and minimum (min_w_) values of the unknown signal and the reference analog to digital conversion range of the YouCare system (±500 mV, range_YC_);
(4) Comparison of the standard deviation (SD_w_) and the variability range (max_w_–min_w_) of the signal to be classified with the reference values (max_t_–min_t_ and SD_t_).

The first block allows the automatic extraction of the reference values of an acceptable signal.

Then, the algorithm was applied to consequent 2 s windows. This period was chosen to be sure that at least one complete beat was present. For each window, maximum (max_w_), minimum (min_w_), and SD (SD_w_) values were extracted.

The third block allows the first primary classification of the signal quality:

(a) If the trace oscillates between values close to saturation ((max_w_–min_w_) > 0.95 × (range_YC_)) (e.g., Figure 1c in a short period (2 s), a short-time significant isoelectric line’s oscillation compromises the reading and the signal is classified as unacceptable;
(b) If the signal’s range (max_w_–min_w_) is lower than 5% of the template’s range (max_t_–min_t_), QRS, P, and T waves are very low or absent. The signal is classified as unacceptable.

The fourth block directly permits comparing the unknown signal and the reference values. The signal is considered unacceptable if the SD_w_ is higher than twice the reference SD_t_. By comparing only the SD values, it is possible to classify a signal as readable, even a signal whose isoelectric line oscillates, without compromising its quality (Figure 1b).

**Figure 2:**
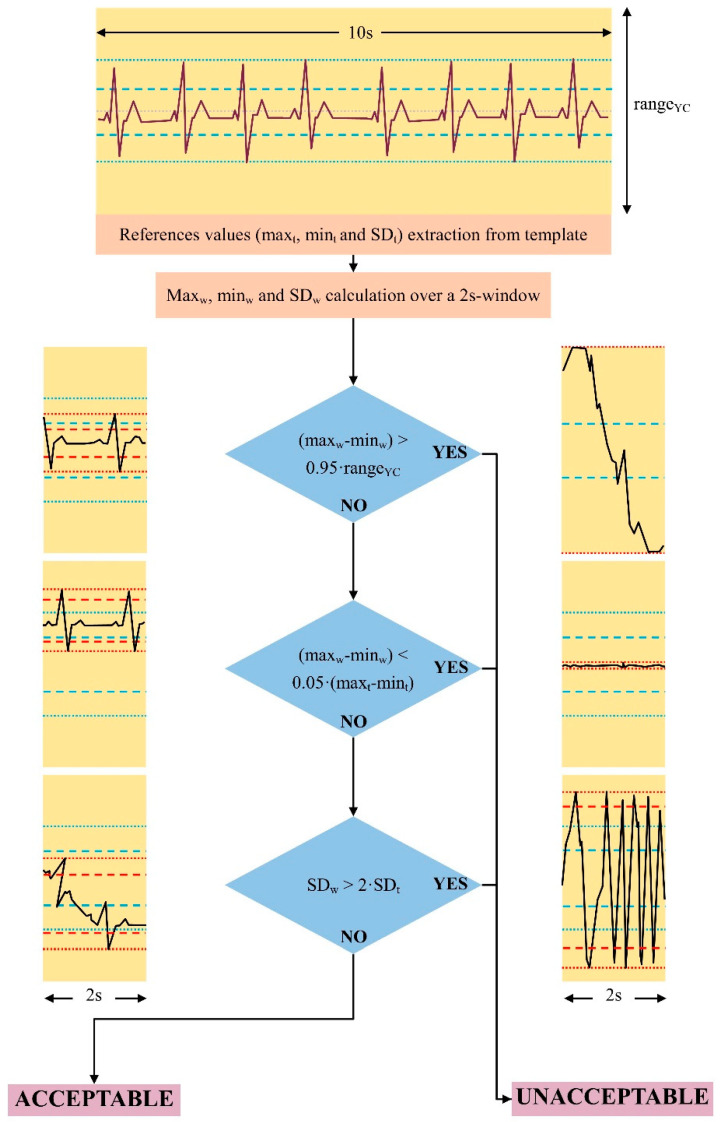
Schematic view of the proposed algorithm. References values are extracted from a stable period of at least 10 s. Then, the algorithm is applied to consecutive ECG 2 s windows, and maximum, minimum, and SD values are extracted. In the first step, the range of variability of the signal (max_w_–min_w_) is compared with the ADC range of the wearable device (range_YC_). If the signal saturates, it is classified as unacceptable. Then, the algorithm verifies and rejects the signal if ECG is absent or with a negligible amplitude. The last step compares the SD of the 2 s windows and the reference template. The signal is classified as unacceptable if the SD_w_ is higher than twice the SD_t_. On the right, some examples of “bad” signals are shown. On the left, some examples of “good” signals are reported. If the ECG is present, even if the baseline is not comparable with the baseline of the reference template, the signal is considered acceptable (blue dotted line: maximum and minimum values of the reference signals; blue dashed lines: mean ± SD of the reference signal; red dotted line: maximum and minimum values of the 2 s window; red dashed lines: mean ± SD of the 2 s window; yellow boxes’ height: ADC range).

Figure 2 shows the algorithm flowchart and some possible cases.

As carried out for the cardiologists’ evaluation, after the 2 s windows analysis performed using the Anscovery System, the classification on 10 s samples was extracted (considering the most prevalent category over this period).

Therefore, the algorithm was tested on a total of 7200 samples. The efficiency of the proposed method was evaluated in terms of sensitivity and specificity [26].

## 3. Results

**Figure 3:**
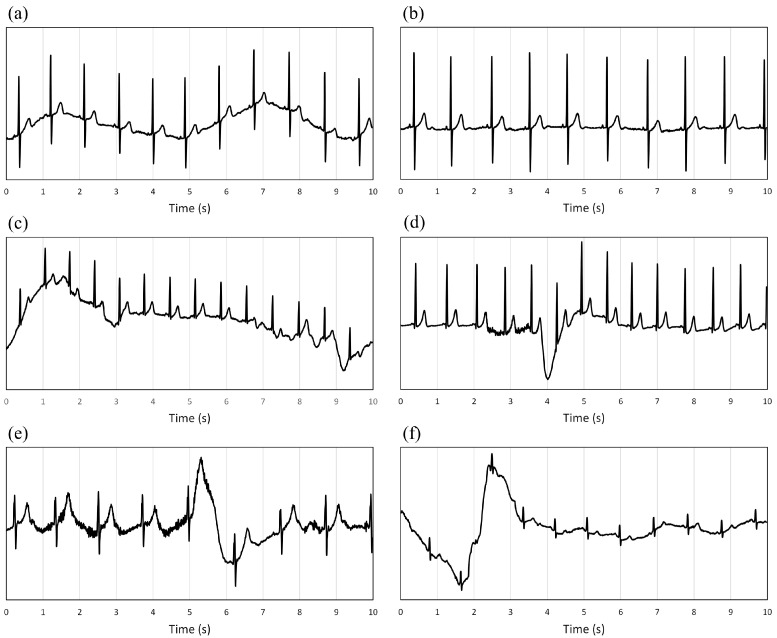
(**a**–**f**): Examples of samples classified as acceptable by the expert cardiologists (the y-scale is different for each plot to make the signal morphology more readable and precise). Classifications were made with a time resolution of a cardiac beat and then reported on 10 s windows considering the predominant type. The consequence is visible in the (**c**–**f**) plots, where the sample was classified as acceptable despite a few seconds of signal with significant oscillations in the isoelectric line.

**Figure 4:**
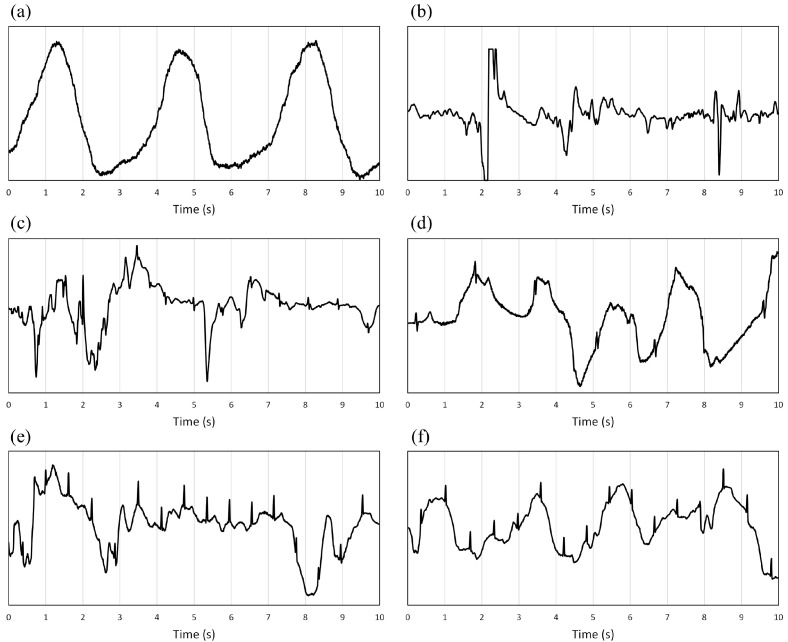
(**a**–**f**): Examples of samples classified as unacceptable by the expert cardiologists (the y-scale is not the same for each plot to make the signal morphology more readable and precise). Classifications were made with a time resolution of a cardiac beat and then reported on 10 s windows considering the predominant type. The consequence is visible in the (**e**,**f**) plots, where despite some beats being visible, they are inserted in a 10 s window with prevalent unreadable parts.

Figure 3 shows samples considered acceptable by cardiologists; Figure 4 shows some examples of unacceptable signals.

A total of 3325 samples (46%) were classified as unacceptable and 3875 (54%) were classified as acceptable. A prevalence of 46% is adequate to test the algorithm’s efficacy.

**Figure 5:**
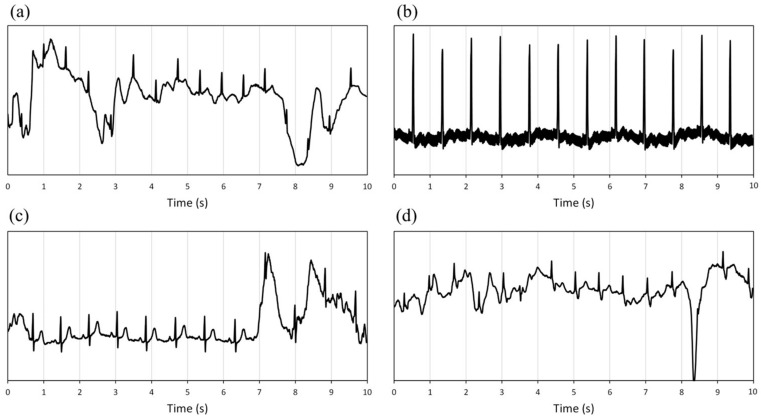
(**a**) Signal classified as unreadable by the cardiologists but acceptable by the algorithm (false negative); (**b**) sample considered not acceptable by cardiologists but acceptable by the algorithm (false negative); (**c**,**d**) signal classified as acceptable by the cardiologists but unreadable by the algorithm.

Figure 5 shows two examples of different classifications by the cardiologists and the algorithm. Figure 5a reports the same signal already present in Figure 4e: for cardiologists, the sample is of bad quality; for the algorithm, the signal is acceptable. This difference depends on the classification of the central part of the sample (from about the 4th to the 7th second) and its length concerning the whole 10 s duration. Since cardiologists put the starting and ending markers by hand with the resolution of 1 beat and the algorithm works on windows of 2 s, even a few seconds of difference in the central part of good sample quality can lead to a different global classification.

Figure 5b shows a different but no less critical mistake: the cardiologists classified the signal with 50 Hz noise as unacceptable, but the algorithm considered it acceptable. This evidence represents a limitation of our approach that does not consider the signal’s harmonic content. Figure 5c,d shows two samples classified as suitable by the cardiologists but not acceptable by the algorithm. In these cases, the misclassification is probably due to baseline oscillation at the samples’ end and duration concerning the time resolution differences between cardiologists and the algorithm.

Table 1 shows the global agreement between each classified sample of 10 s extracted from the 20 recordings of 1 *h*-ECG. The algorithm presents a global efficiency of 95.1%, calculated as the total number of correct classifications over the total number of considered samples (7200).

The algorithm presents a sensitivity of 94.7% (with a 95% confidence interval of 0.8%) and a specificity of 95.4% (95%CI: ±0.7%). The positive and negative predictive values (PPV and NPV) are 94.7% and 95.5%, respectively, and confirm the algorithm’s efficacy for ECG quality classification.

The positive and negative likelihood ratios (LR+ and LR−) are 20.7 and 0.055, respectively.

Among the 20 patients, the global algorithm efficiency ranges between 0.89 and 1.00, with a median value of 0.95, equal to the mean value.

## 4. Discussion

The transition to a more widespread and inclusive healthcare system, reaching all those in need, must necessarily involve two equally crucial successive steps: firstly, education on the use of simple yet efficient medical devices; secondly, and no less critically, wearable devices must be able to address issues arising from the poor quality of recorded signals [27]. In practice, continuous but flawed monitoring would make the device’s use futile, nullifying the time and money invested up to that point. Therefore, cost reduction must be effective, and the mobile devices that acquire relevant physiological signals must work correctly and be reliable [8,28,29,30]. Paradoxically, the instrument ensuring the best acquisition technology with advanced analysis systems is often the most expensive. This statement poses an unsolvable question: how do we reduce costs while maintaining good-quality recorded information? We propose to equip acquisition devices with a simple and economical method for recognizing artifacts and noise (due to electrode detachment and movements), improving the signal quality essential for clinical assessment. Quality control must be performed during acquisition, allowing the wearer or the remote monitoring technician to intervene promptly to restore optimal quality. Without timely intervention, there is a risk of acquiring clinically unusable signals.

In this study, we focused on the ECG signal, probably the most interesting clinical parameter for which most devices on the market are equipped. Electrocardiographic monitoring is crucial for detecting arrhythmias (e.g., atrial fibrillation, ventricular tachycardia or bradycardia, bundle branch blocks, etc.) [31]. The ECG quality is vital for signal processing, which is a fundamental step in QRS detection [32], and for signal interpretation by medical doctors or computers. The proposed algorithm can work in real time (with a few seconds’ delay) and immediately assess the acquired signal’s quality. This feedback, for example, in the form of a sound alarm, could assist the device user or the remote monitoring technician in correcting hardware issues and improving signal acquisition. We are aware that our approach is “invasive” and may be disruptive for the individual, especially during phases of life where artifacts are “necessary” (e.g., during movement). But, when the device is used for clinical screening and monitoring, the potential data loss could make it impossible for the physician to evaluate the ECG signal. In these cases, auditory and visual feedback are crucial to help the device and restore adequate quality. As is often the case, it is a trade-off: on the one hand, total freedom of movement, with the risk of rendering the acquisition useless and wasting money, and on the other, a more conscious procedure that may become intrusive in the patient’s daily life but would allow effective monitoring. If the device’s use aims to reduce disparities and reach as many people as possible, a slight inconvenience for the individual can and should be tolerated to achieve the desired goal. As a demonstration of these statements, it is interesting to highlight that in the present paper, the total number of samples considered not acceptable by expert cardiologists is about 46%: this percentage suggests that the presence of artifacts can be very invasive and makes an essential percentage of the acquired ECG noisy and not usable for clinical purposes. A solution to this problem is mandatory to make these wearables perform effectively.

The proposed algorithm is simple and undoubtedly basic compared to what is available today, often combined with sophisticated and expensive devices. We do not employ advanced methods (for example, artificial intelligence approaches) but analyze only the mean, range of variability, and standard deviation.

The algorithm was blindly tested on ECG signals acquired using a commercial device (YouCare, AccYouRate). Since this research aims to validate an algorithm, population characteristics (i.e., age, sex, pathologies) were not considered, since they did not constitute a discriminant for the final analysis.

Samples were classified as “good” with P, QRS, and T visible. The P wave can be negligible if a diagnosis of atrial fibrillation is present or performed by the cardiologist. Only the visibility of P, QRS, and T waves does not always mean the possibility of measuring such parameters as ST elevation, and this can be considered a limit of our approach. However, the balance between benefits and costs was kept tending towards “minimal benefits”; otherwise, a larger quantity of samples would be rejected, reducing the possibility even for an essential (but valuable) evaluation. This issue is critical in contexts where expensive devices are absent, and wearables can be an excellent alternative to them, allowing a clinical evaluation despite the lack of funding. The algorithm’s application to a cohort of 7200 samples demonstrated an overall effectiveness of 95.1%, with high sensitivity and specificity (94.7% and 95.4%). More sophisticated algorithms exist [18] but are undoubtedly more complex and costly.

On the other hand, a sensitivity and specificity of around 95% are excellent and would allow efficient, targeted intervention to solve the problem. Even if the algorithm wrongly considered 5% of cases readable when the signal is genuinely unusable, it would be a short period and undoubtedly not crucial for clinical evaluation. PPV, NPV, LR+, and LR- values confirm the reliability of our proposal, considering a prevalence of unacceptable samples of 46%. Moreover, the algorithm efficiency ranges from 0.89 to 1.00. Even if the signal is more difficult to classify, the algorithm performance can be considered acceptable.

Additionally, the classifications by cardiologists and the algorithm were subsampled over 10 s windows for the final comparison. This choice was made to make the obtained results comparable. While better temporal resolution is always desirable and may have ensured a better outcome (as illustrated in the examples in Figure 5), considering the perspective of using the algorithm as a real-time classifier, frequent acoustic or light feedback would render the acquisition device unusable. For a practical application of the algorithm, 10 s windows are too short and should be extended to 1 min intervals or more. The processing time needed for the algorithm to analyze 1 h of signal entirely was less than 5 s. Implementing the method in real-time means that the mean, max, and SD can be calculated at each sample acquisition. Classification results can be available with the delay of the chosen window length (in the paper, 10 s). The proposed algorithm needs to be improved in correctly identifying 50 Hz noise, as illustrated in Figure 5b. Electromagnetic noise could be caused by poor electrode placement and inadequate shielding against external electromagnetic fields that interfere with the device. Fortunately, this network noise seems to occur in only one patient, suggesting that it is rare. It is worth noting that despite this limitation, the algorithm can still detect QRS complexes, enabling an assessment of heart rhythm variability, even if the P and T waves cannot be accurately identified. A possible solution can be to extract the frequency spectrum for each considered window and to evaluate the amplitude of the 50 Hz harmonic compared to the same value of the clean reference signal.

## 5. Conclusions

In a healthcare context where lower-performing and low-cost sensors are often used instead of much more expensive medical equipment, the subject undergoing evaluation must be active in collecting signals of biomedical interest. It is impossible to hope that a passive attitude will lead to reliable and usable results in a clinical context. The acquisition of a clean ECG signal is essential for diagnosis, and the presence of noise or artifacts due to the movement or positioning of the electrodes makes monitoring useless and, thus, generates a significant economic loss.

The proposed algorithm represents an excellent solution for the real-time classification of ECG quality. It can be implemented with economical and computationally meager costs in all commercially available devices. If the wearable device is connected to a smartphone (as in our experimental setup), algorithm embedding can be elementary, and further improvements (for example, visual feedback) can be made.

In conclusion, despite further investigations being necessary to validate the algorithm in real time, the results are practical and encouraging.

## Figures and Tables

**Table 1:** Agreement between classifications.

	Classification by Cardiologists			
Classification by algorithm		Not acceptable	Acceptable	Total
	Not acceptable	3150 (94.7%)	177 (4.6%)	3327
	Acceptable	175 (5.3%)	3698 (95.4%)	3873

## Data Availability

The data presented in this study are available on request from the corresponding author. The data are not publicly available due to the privacy policies of the Ethics Committee Area Vasta Emilia Centro—CE-AVEC—Bologna.
